# Severe postoperative complications after minimally invasive esophagectomy reduce the long-term prognosis of well-immunonutrition patients with locally advanced esophageal squamous cell carcinoma

**DOI:** 10.1080/07853890.2024.2440622

**Published:** 2024-12-13

**Authors:** Chao Chen, Shao-Jun Xu, Zhi-Fan Zhang, Cheng-Xiong You, Yun-Fan Luo, Rui-Qin Chen, Shu-Chen Chen

**Affiliations:** a Department of Thoracic Surgery, Fujian Medical University Union Hospital, Fuzhou, Fujian Province, China; b Key Laboratory of Ministry of Education for Gastrointestinal Cancer, Fujian Medical University, Fuzhou, Fujian Province, China; c Fujian Provincial Key Laboratory of Cardiothoracic Surgery, Fujian Medical University Union Hospital, Fuzhou, Fujian Province, China

**Keywords:** Minimally invasive esophagectomy, esophageal squamous cell carcinoma, severe postoperative complications, prognostic nutritional index

## Abstract

**Background:**

While severe postoperative complications (SPCs) impact cancer prognosis, their effect on locally advanced esophageal squamous cell carcinoma (ESCC) patients with varying immunonutritional statuses after minimally invasive esophagectomy (MIE) is unclear.

**Methods:**

This retrospective study analyzed 442 patients with locally advanced ESCC who underwent MIE, investigating the relationship between SPCs and survival based on preoperative immunonutritional status, determined by the prognostic nutritional index (PNI). Nomograms were developed for patients with preserved immunonutritional status using Cox regression, and their performance was assessed.

**Results:**

Of the patients, 102 (23.1%) experienced SPCs after MIE. Five-year overall survival (OS) and disease-free survival (DFS) were significantly different between SPCs and non-SPCs groups (*p* < 0.001). In the preserved immunonutritional group, SPCs significantly reduced 5-year OS (*p* = 0.008) and DFS (*p* = 0.011), but not in the poor immunonutritional group (OS *p* = 0.152, DFS *p* = 0.098). Multivariate Cox regression identified SPCs as an independent risk factor for OS (HR = 1.653, *p* = 0.013) and DFS (HR = 1.476, *p* = 0.039). A nomogram for predicting OS and DFS in preserved immunonutritional patients demonstrated excellent performance.

**Conclusions:**

SPCs significantly affect prognosis in ESCC patients with preserved immunonutritional status after MIE. Nomograms based on SPCs can predict OS and DFS in these patients.

## 1. Introduction

Esophageal carcinoma is the seventh most prevalent malignant neoplasm globally with the sixth highest number of cancer-related fatalities [1]. Esophageal cancer is an aggressive and highly malignant tumour with an early onset of lymph node metastasis. However, early esophageal cancer has atypical symptoms. As a result, most esophageal cancer patients with progressive dysphagia are often diagnosed in the middle or late stages, resulting in poor prognosis [2]. Besides, the 5-year postoperative survival rates of esophageal cancer patients are different among the groups (group based on the tumour-lymph node-metastasis (TNM) staging system, established by the American Joint Committee on Cancer (AJCC)). Specifically, the survival rates at stages IIa, IIb, III, and IV are 44.2%, 42.8%, 17.1%, and 13.2%, respectively [3,4].

Definitive surgical intervention combined with lymph node dissection has been widely used for the management of esophageal carcinoma. Besides, minimally invasive esophagectomy (MIE) has also been used for esophageal carcinoma treatment due to evolution of surgical techniques [5]. However, most esophageal cancer patients (50%) experience severe postoperative complications (SPCs) after minimally invasive procedures [6]. Besides increased treatment costs and prolonged hospitalization, SPCs influences the postoperative convalescence and quality of life of esophageal cancer resection recipients. SPCs also affect the long-term survival prospects of esophageal cancer resection recipients [7].

Previous studies have found that SPCs have different effects on the prognosis of tumour patients under different immune and nutritional status [8–10]. A range of nutritional and immune markers, including the prognostic nutrition index (PNI), Glasgow prognostic score, and neutrophil-albumin ratio, can be used to identify prognostic survival in cancer patients [11–15]. PNI was initially defined by Buzby et al. and it encompasses multiple parameters [16]. PNI assesses the risk of postoperative complications based on a patient’s initial nutritional status. The PNI is widely used to indicate immune-nutritional status. Notably, PNI is significantly correlated with treatment efficacy and long-term prognosis in various solid tumour types [17–19]. Esophageal squamous cell carcinoma (ESCC) is the dominant histopathological subtype of esophageal cancer in Asia, representing 90% of all cases [20]. Nevertheless, the impact of SPCs on the prognosis of locally advanced ESCC patients with distinct immune-nutritional statuses is unclear.

This study aimed to assess the preoperative immune-nutritional status of patients with locally advanced ESCC using the PNI and the effect of SPCs on the long-term prognosis of patients with varying immune-nutritional statuses after MIE.

## 2. Materials and methods

### 2.1. Study populations and design

This retrospective analysis involved a continuous cohort of patients with locally advanced ESCC who had undergone MIE at the Fujian Medical University Affiliated Union Hospital from January 2011 to December 2018. Inclusion criteria were: (1) Patients with histologically-confirmed ESCC, (2) postoperative pathological stage II-III patients, (3) Patients who underwent MIE, and (4) Patients without concurrent malignancies. Exclusion criteria included: (1) Patients with postoperative pathological evidence of positive margins, (2) Patients whose intraoperative findings showed tumour metastasis, (3) Patients with incomplete clinical data or those lost to follow-up, (4) patients who changed to open surgery, and (5) individuals with severe cardiopulmonary insufficiency and could not tolerate surgery. Finally, 442 patients with locally advanced ESCC were enrolled in this study. The patients were divided into groups based on the presence or absence of SPCs. Clinical and pathological data, surgical details, and postoperative complications were also recorded. Pathological diagnoses and staging were performed following the 8th edition of the TNM staging system developed by the AJCC. This study followed the Helsinki Declaration protocols and was approved by the Institutional Review Board of Fujian Medical University Union Hospital (IRB number: 2021QH009). The requirement for written informed consent was waived by the ethics committee of Fujian Medical University Union Hospital because of the retrospective nature of the study.

### 2.2. Definitions

The postoperative severe complications were classified based on the Clavien-Dindo grading system, where grade III and higher complications were regarded as Severe Postoperative Complications (SPCs). The Prognostic Nutritional Index (PNI) was determined as described by Onodera (the multiplication of the serum albumin level (g/100 ml) by 10 and the total peripheral lymphocyte count (10^9^/mm^3^) by 0.005) [8]. The patients were categorized into two groups, with the lower quartile Q1 as the truncation point (poor immune-nutritional group and preserved immune-nutritional group).

### 2.3. Follow-up

Comprehensive outpatient follow-up assessments, including physical, blood biochemical, and imaging examinations, were scheduled 1, 3, and 6 months after the operation, followed by biannual reassessments for 2–3 years. The follow-ups were then conducted semi-annually for 5 years. Notably, follow-up was conducted *via* telephone or written communication for patients who could not attend outpatient follow-up evaluations.

### 2.4. Outcome measures

The primary outcome of this study was to analyze the effect of SPC on the prognosis of ESCC patients in different immunonutritional states, and to evaluate independent risk factors affecting Overall Survival (OS) and Disease-Free Survival (DFS). The secondary outcome was to establish and internally validate a nomogram for predicting the prognosis of ESCC patients for clinical convenience.

### 2.5. Treatment protocols

All patients received a complete preoperative assessment after admission, including a detailed medical history and physical examination by the resident, and laboratory tests such as blood, urine, and stool. Gastroscopy, enhanced computed tomography of the neck, chest and upper abdomen and ultrasound of the neck, as well as cardiopulmonary function assessment. Patients are screened for nutrition before surgery, and those at risk of nutrition are given nutritional preparations by oral or enteral nutrition.

Patients with locally advanced esophageal cancer assessed as cT3-4a or N1-2 may be considered for preoperative neoadjuvant chemotherapy [21]. Neoadjuvant therapy is applied based on a comprehensive assessment of the patient’s physical condition, doctor’s evaluation, and patient’s willingness. The neoadjuvant treatment options in this study include chemotherapy, radiotherapy, or a combination of both. According to the recommendations in the diagnosis and treatment guidelines for esophageal cancer, we recommend postoperative adjuvant therapy for pT4aN0M0/PT1-4aN + M0 ESCC patients [22]. Postoperative adjuvant treatment is not mandatory, but is applied based on a comprehensive assessment of pathological findings, treatment preferences, physical condition, and physician assessment.

The surgical method, approach and procedure are determined by the surgeon. Lymph node dissection is mainly determined by the location of the tumour: if there is no suspicious enlarged lymph node in the neck, the middle and lower thoracic esophageal cancer is routinely treated with complete thoracic and abdominal two-field lymph node dissection. If there is suspicious enlarged lymph node in the neck and upper thoracic esophageal cancer, three-field lymph node dissection of the neck, chest and abdomen is performed.

After the operation, the patient is given fasting, continuous gastrointestinal decompression, total intravenous nutrition and other treatments. Intravenous nutrition is gradually reduced until intravenous nutrition is stopped. When it is confirmed that there is no anastomotic leakage and then a liquid diet is started and gradually transitioned to a semi-liquid diet.

### 2.6. Statistical analysis

The OS was defined as the duration from the surgical intervention to the occurrence of death or the latest follow-up. The DFS was defined as the time from the surgical procedure to the emergence of tumour recurrence, metastasis, the ultimate follow-up visit, or death due to any cause.

χ2 test or Fisher’s exact test was used to analyze categorical variables, while the t-test or Mann-Whitney U test was used for the analysis of continuous variables. Kaplan-Meier method was used to compare OS and DFS between groups. The disparities in survival were assessed *via* the Log-rank test. The factors influencing OS and DFS were evaluated through univariate and multivariate Cox proportional hazard regression, followed by the construction of nomograms based on the multifactorial outcomes. The performance of the predictive model was assessed through internal validation *via* 1000 iterations of the Bootstrap resampling technique, thereby generating calibration curves for model validation. The X-Tile software (Version 3.6.1) and an ‘enumeration method’, were used to determine the optimal cutoff values for survival risk scoring. The OS and DFS risk scores were grouped into three tiers: low risk, moderate risk, and high risk.

IBM SPSS Version 25.0 and R Version 3.5.1 were used for all statistical analyses. *p* < 0.05 was considered statistically significant level.

## 3. Results

### 3.1. Postoperative complications

About 23.1% (102 of 402 cases) of patients experienced SPCs postoperatively, including anastomotic leakage (*n* = 70; 68.6%), pulmonary infections/pneumothorax (*n* = 68; 66.7%), acute respiratory distress syndrome (*n* = 10; 9.8%), postoperative hemorrhage (*n* = 6 cases; 5.9%), chylothorax (*n* = 9; 8.8%), and empyema (*n* = 4; 3.9%) (Figure S1).

### 3.2. Clinicopathological characteristics

In the study population, 94(21.3%) patients were older than 65 years, and 358 (81.0%) patients had no comorbidities. In addition, 62(14.0%) patients were assessed as high-risk malnutrition by MUST. In the included population, the majority of (69.9%) patients were male. There were 78(17.6%) patients receiving neoadjuvant therapy, 183 (41.4%) patients receiving postoperative adjuvant therapy, and 102(23.1%) patients developing SPCs after surgery (Table S1).

The pathological characteristics of the SPCs group and the non-SPCs group are presented in Table 1. The SPCs group had 102 patients (mean age; 60 ± 8.1 years), while the non-SPCs group comprised 340 patients (average age; 59 ± 8.3 years). Notably, compared with the SPCs group, the non-SPCs group had a higher proportion of female patients (*p* = 0.008), higher ASA score (*p* < 0.001), and lower tumour invasion (*p* = 0.04). However, age (*p* = 0.932), BMI (*p* = 0.163), comorbidities (*p* = 0.297), MUST (*p* = 0.168), tumour location (*p* = 0.902), histologic grade (*p* = 0.188), lymph node metastasis (*p* = 0.421), neoadjuvant therapy (*p* = 0.374), surgical method (*p* = 0.609), lymphadenectomy (*p* = 0.135), intraoperatve blood loss (*p* = 0.695), adjuvant therapy (*p* = 0.958) and PNI (*p* = 0.609) were not significantly ­different between the SPCs and non-SPCs groups.

**Table 1.** Correlations between SPCs and clinicopathological factors in patient with locally advanced ESCC.

Characteristics	SPCs		*P* value
	Yes(n = 102)	No(n= 340)	
**Age(years)**			0.932
≤65	80(78.4%)	268(78.8%)	
>65	22(21.6%)	72(21.2%)	
**Sex**			0.008
Female	20(19.6%)	113(33.2%)	
Male	82(80.4%)	227(66.8%)	
**BMI (kg/m^2^)**			0.163
≤18.5	7(6.9%)	41(12.1%)	
18.5–25	85(83.3%)	253(74.4%)	
≥25	10(9.8%)	46(13.5%)	
**Comorbidities**			0.297
None	88(86.3%)	270(79.4%)	
Hypertension	9(8.8%)	56(16.5%)	
Diabetes	4(3.9%)	11(3.2%)	
Coronary heart disease	1(1.0%)	3(0.9%)	
**MUST**			0.168
Low risk	74(72.5%)	273(80.3%)	
Medium risk	8(7.8%)	25(7.4%)	
High risk	20(19.6%)	42(12.4%)	
**ASA score**			
I/II	65(63.7%)	300(88.2%)	<0.001
III/IV	37(36.3%)	40(11.8%)	
**Tumour location**			0.902
Proximal	10(9.8%)	34(10.0%)	
Mid	66(64.7%)	212(62.4%)	
Distal	26(25.5%)	94(27.6%)	
**Histologic grade**			0.188
Gx/G1	46(45.1%)	120(35.3%)	
G2	43(42.2%)	174(51.2%)	
G3	13(12.7%)	46(13.5%)	
**Tumour invasion**			0.04
T1	2(2.0%)	30(8.8%)	
T2	16(15.7%)	63(18.5%)	
T3/T4a	84(82.4%)	247(72.6%)	
**Lymph node metastasis**			0.421
N0	37(36.3%)	119(35.0%)	
N1	28(27.5%)	120(35.3%)	
N2	31(30.4%)	81(23.8%)	
N3	6(5.9%)	20(5.9%)	
**Neoadjuvant therapy**			0.374
Yes	21(20.6%)	57(16.8%)	
No	81(79.4%)	283(83.2%)	
**Surgical method**			0.609
McKeown	90(88.2%)	306(90.0%)	
Ivor Lewis	12(11.8%)	34(10.0%)	
**Lymphadenectom**y			0.135
Two-field	86(84.3%)	305(89.7%)	
Three-field	16(15.7%)	35(10.3%)	
**Intraoperatve blood loss(ml)**			0.695
≤100	48(47.1%)	175(51.5%)	
100–200	38(37.3%)	120(35.3%)	
≥200	16(15.7%)	45(13.2%)	
**Adjuvant therapy**			0.958
No	60(58.8%)	199(58.5%)	
Yes	42(41.2%)	141(41.5%)	
**PNI**			0.609
≥47.1	73(71.6%)	252(74.1%)	
<47.1	29(28.4%)	88(25.9%)	

### 3.3. Relationship between SPCs and long-term outcome

**Figure 1.**
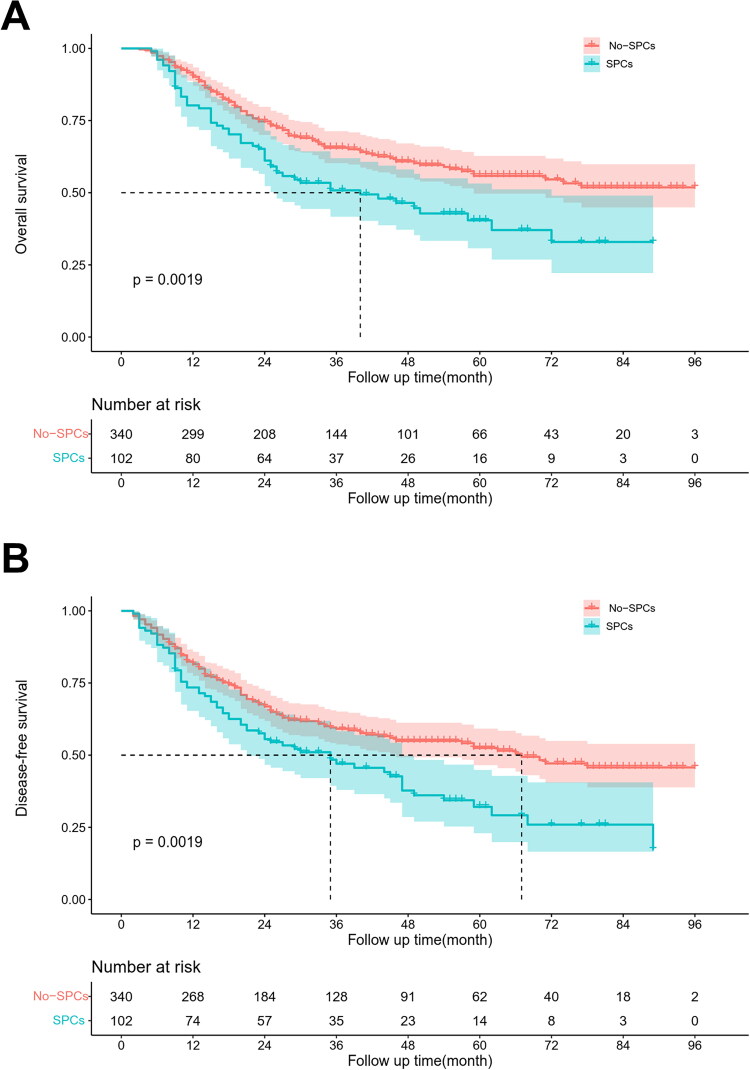
Kaplan–Meier survival curves for OS (A) and DFS (B) in locally advanced ESCC patients with SPCs and non-SPCs.

Kaplan-Meier survival analysis revealed that the 5-year OS rates for the SPCs group and the non-SPCs group were 40.4% and 55.8%, respectively (*p* < 0.001), while the 5-year DFS rates were 32.1% and 52.5%, respectively (*p* < 0.001). Compared with the non-SPCs group, the 5-year OS (*p* = 0.0019) and DFS (*p* = 0.0019) were significantly reduced in the SPCs group (Figure 1). These findings indicate that SPCs is a key predictive factor for patients with locally progressed ESCC. Moreover, univariate and multivariate Cox regression analyses showed that N stage, intraoperative hemorrhage volume, and SPCs are the autonomous risk factors impacting both OS and DFS of patients with locally advanced ESCC (Table 2).

**Table 2.** Univariate and multivariate Cox analysis of clinicopathological factors for OS and DFS.

Characteristics	Overall survival				Disease-free survival			
	Univariable analysis		Multivariable analysis		Univariable analysis		Multivariable analysis	
	HR(95%CI)	*P*-value	HR(95%CI)	*P*-value	HR(95%CI)	*P*-value	HR(95%CI)	*P*-value
**Age(years)**								
≤65								
>65	1.322(0.937–1.865)	0.112			1.292(0.937–1.782)	0.119		
**Sex**								
Female								
Male	1.088(0.777–1.524)	0.624			0.996(0.726–1.365)	0.978		
**BMI (kg/m^2^)**								
≤18.5								
18.5–25	0.993(0.620–1.589)	0.977			1.094(0.693–1.727)	0.699		
≥25	1.166(0.644–2.112)	0.612			1.425(0.815–2.491)	0.214		
**Comorbidities**								
None								
Hypertension	0.766(0.489–1.198)	0.243			0.681(0.441–1.052)	0.083		
Diabetes	0.536(0.199–1.448)	0.219			0.563(0.231–1.370)	0.205		
Coronary heart disease	1.992(0.493–8.048)	0.334			1.469(0.364–5.927)	0.589		
**MUST**								
Low risk								
Medium risk	1.154(0.621–2.145)	0.651	1.063(0.561–2.013)	0.852	1.109(0.627–1.960)	0.723		
High risk	1.470(1.004–2.154)	0.048	1.366(0.914–2.042)	0.128	1.389(0.966–1.996)	0.076		
**ASA score**								
I/II								
III/IV	1.636(1.121–2.387)	0.011	1.232(0.810–1.876)	0.33	1.536(1.076–2.193)	0.018	1.241(0.851–1.809)	0.262
**Tumour location**								
Proximal								
Mid	1.346(0.786–2.305)	0.279			1.637(0.960–2.790)	0.07		
Distal	1.198(0.669–2.147)	0.543			1.402(0.790–2.487)	0.248		
**Histologic grade**								
Gx/G1								
G2	1.089(0.792–1.497)	0.6			1.118(0.832–1.503)	0.459		
G3	1.221(0.753–1.978)	0.418			1.152(0.733–1.810)	0.54		
**Tumour invasion**								
T1								
T2	0.914(0.437–1.912)	0.812			0.981(0.488–1.972)	0.957		
T3/T4a	1.547(0.815–2.938)	0.182			1.652(0.897–3.042)	0.107		
**Lymph node metastasis**								
N0								
N1	1.492(1.001–2.224)	<0.001	1.540(1.025–2.312)	0.038	1.407(0.976–2.028)	0.067	1.406(0.969–2.039)	0.072
N2	2.301(1.557–3.401)	<0.001	2.509(1.681–3.743)	<0.001	2.303(1.612–3.289)	<0.001	2.461(1.711–3.540)	<0.001
N3	4.427(2.546–7.697)	<0.001	5.215(2.952–9.211)	<0.001	3.957(2.310–6.780)	<0.001	4.743(2.584–7.744)	<0.001
**Neoadjuvant therapy**								
Yes								
No	1.374(0.976–1.936)	0.069			1.344(0.976–1.851)	0.07		
**Surgical method**								
McKeown								
Ivor Lewis	0.682(0.413–1.126)	0.135			0.685(0.432–1.089)	0.11		
**Lymphadenectomy**								
Two-field								
Three-field	0.897(0.577–1.393)	0.627			0.875(0.574–1.335)	0.537		
**Intraoperatve blood loss(ml)**								
≤100								
100–200	1.245(0.902–1.717)	0.182	1.235(0.893–1.708)	0.202	1.159(0.857–1.568)	0.338	1.139(0.841–1.542)	0.401
≥200	2.003(1.293–3.104)	0.002	2.236(1.423–3.514)	<0.001	2.101(1.416–3.118)	<0.001	2.304(1.534–3.461)	<0.001
**Adjuvant therapy**								
No								
Yes	0.696(0.509–0.952)	0.023	0.777(0.565–1.068)	0.121	0.616(0.459–0.826)	0.001	0.659(0.490–0.887)	0.006
**PNI**								
≥47.1								
<47.1	0.913(0.655–1.273)	0.593			0.943(0.694–1.281)	0.709		
**SPCs**								
No								
Yes	1.639(1.193–2.250)	0.002	1.591(1.135–2.231)	0.007	1.585(1.179–2.130)	0.002	1.500(1.097–2.049)	0.011

### 3.4. The correlation between SPCs and clinicopathological factors based on PNI levels

The correlation between distinct immune-nutritional statuses and the clinical-pathological characteristics related to SPCs is shown in Table 3. In the poor immunenutritional group, the ASA score was significantly different between the SPCs group and non-SPCs group (*p* = 0.003). However, age (*p* = 0.280), sex (*p* = 0.287), BMI (*p* = 0.626), comorbidities (*p* = 0.700), MUST (*p* = 0.672), tumour location (*p* = 0.385), histologic grade (*p* = 0.515), tumour invasion (*p* = 0.250), lymph node metastasis (*p* = 0.617), neoadjuvant therapy (*p* = 0.808), surgical method (*p* = 0.249), lymphadenectomy (*p* = 0.302), intraoperatve blood loss (*p* = 0.778) and adjuvant therapy (*p* = 0.997) were not significantly different between the SPCs group and non-SPCs group.

**Table 3.** Correlations between SPCs and clinicopathological factors in the high and low PNI groups.

Characteristics	PNI < 47.1			PNI ≥ 47.1		
	SPCs			SPCs		
	Yes (*n* = 25)	No(*n* = 84)	P value	Yes (*n* = 77)	No(*n* = 256)	P value
**Age(years)**			0.280			0.553
≤65	16(64.0%)	63(75.0%)		64(83.1%)	205(80.1%)	
>65	9(36.0%)	21(25.0%)		13(16.9%)	51(19.9%)	
**Sex**			0.287			0.015
Female	5(20.0%)	26(31.0%)		62(80.5%)	169(66.0%)	
Male	20(80.0%)	58(69.0%)		15(19.5%)	87(34.0%)	
**BMI (kg/m^2^)**			0.626			0.260
≤18.5	2(8.0%)	12(14.3%)		5(6.5%)	29(11.3%)	
18.5–25	21(84.0%)	63(75.0%)		64(83.1%)	190(74.2%)	
≥25	2(8.0%)	9(10.7%)		8(10.4%)	37(14.5%)	
**Comorbidities**			0.700			
None	20(80.0%)	70(83.3%)		68(88.3%)	200(78.1%)	0.099
Hypertension	5(20.0%)	14(16.7%)		4(5.2%)	42(16.4%)	
Diabetes	0(0.0%)	0(0.0%)		4(5.2%)	11(4.3%)	
Coronary heart disease	0(0.0%)	0(0.0%)		1(1.3%)	3(1.2%)	
**MUST**			0.672			0.248
Low risk	18(72.0%)	67(79.8%)		56(72.7%)	206(80.5%)	
Medium risk	2(8.0%)	6(7.1%)		6(7.8%)	19(7.4%)	
High risk	5(20.0%)	11(13.1%)		15(19.5%)	31(12.1%)	
**ASA score**			0.003			<0.001
I/II	17(68.0%)	77(91.7%)		48(62.3%)	223(87.1%)	
III/IV	8(32.0%)	7(8.3%)		29(37.7%)	33(12.9%)	
**Tumour location**			0.385			0.969
Proximal	1(4.0%)	5(6.0%)		9(11.7%)	29(11.3%)	
Mid	20(80.0%)	55(65.5%)		46(59.7%)	157(61.3%)	
Distal	4(16.0%)	24(28.6%)		22(28.6%)	70(27.3%)	
**Histologic grade**			0.515			0.325
Gx/G1	12(48.0%)	31(36.9%)		34(44.2%)	89(34.8%)	
G2	9(36.0%)	41(48.8%)		34(44.2%)	133(52.0%)	
G3	4(16.0%)	12(14.3%)		9(11.7%)	34(13.3%)	
**Tumour invasion**			0.250			0.137
T1	0(0.0%)	7(8.3%)		2(2.6%)	23(9.0%)	
T2	3(12.0%)	14(16.7%)		13(16.9%)	49(19.1%)	
T3/T4a	22(88.0%)	63(75.0%)		62(80.5%)	184(71.9%)	
**Lymph node metastasis**			0.617			0.465
N0	10(40.0%)	26(31.0%)		27(35.1%)	93(36.3%)	
N1	6(24.0%)	31(36.9%)		22(28.6%)	89(34.8%)	
N2	7(28.0%)	23(27.4%)		24(31.2%)	58(22.7%)	
N3	2(8.0%)	4(4.8%)		4(5.2%)	16(6.3%)	
**Neoadjuvant therapy**			0.808			0.375
Yes	5(20.0%)	15(17.9%)		16(20.8%)	42(16.4%)	
No	20(80.0%)	69(82.1%)		61(79.2%)	214(83.6%)	
**Surgical method**			0.249			0.218
McKeown	24(96.0%)	74(88.1%)		66(85.7%)	232(90.6%)	
Ivor Lewis	1(4.0%)	10(11.9%)		11(14.3%)	24(9.4%)	
**Lymphadenectomy**			0.302			0.263
Two-field	20(80.0%)	74(88.1%)		66(85.7%)	231(90.2%)	
Three-field	5(20.0%)	10(11.9%)		11(14.3%)	25(9.8%)	
**Intraoperatve blood loss(ml)**			0.778			0.413
≤100	13(52.0%)	37(44.0%)		35(45.5%)	138(53.9%)	
100–200	9(36.0%)	36(42.9%)		29(37.7%)	84(32.8%)	
≥200	3(12.0%)	11(13.1%)		13(16.9%)	34(13.3%)	
**Adjuvant therapy**			0.997			0.954
No	14(56.0%)	47(56.0%)		46(59.7%)	152(59.4%)	
Yes	11(44.0%)	37(44.0%)		31(40.3%)	104(40.6%)	

For the preserved immunenutritional group, the non-SPCs group had a higher proportion of male patients (*p* = 0.015) and lower ASA score (*p* < 0.001) than the SPCs group. However, age (*p* = 0.553), BMI (*p* = 0.260), comorbidities (*p* = 0.099), MUST (*p* = 0.248), tumour location (*p* = 0.969), histologic grade (*p* = 0.325), tumour ­invasion (*p* = 0.137), lymph node metastasis (*p* = 0.465), neoadjuvant therapy (*p* = 0.375), surgical method (*p* = 0.218), lymphadenectomy (*p* = 0.263), intraoperatve blood loss (*p* = 0.413) and adjuvant therapy (*p* = 0.954) were not significantly different between the SPCs group and non-SPCs group.

### 3.5. The relationship between SPCs and survival based on PNI levels

**Figure 2.**
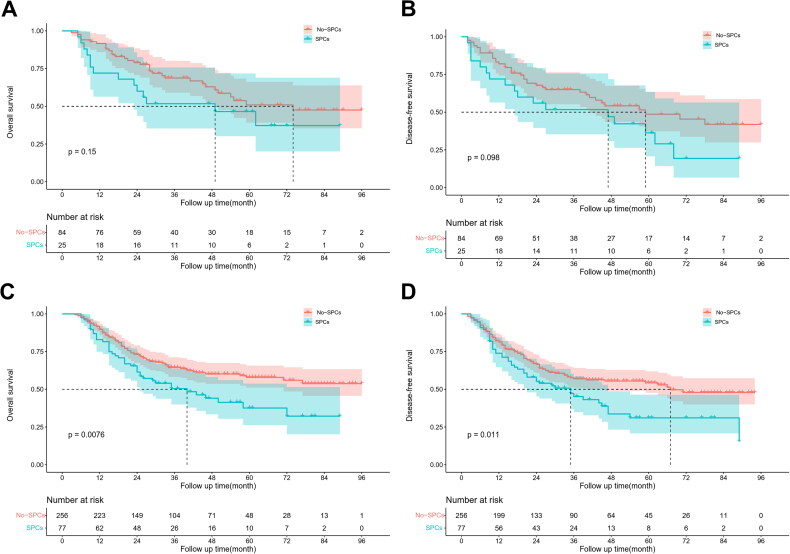
Kaplan–Meier survival curves for patients with SPCs and non-SPCs. OS (A) and DFS (B) in low-PNI; OS (C) and DFS (D) in high-PNI.

Kaplan-Meier survival analysis showed that SPCs significantly reduced 5-year OS (57.9% vs. 37.6%, *p* = 0.008) and 5-year DFS (54.3% vs. 31.1%, *p* = 0.011) of locally advanced ESCC patients with preserved immune-nutritional status. However, SPCs did not significantly affect 5-year OS (50.9% vs. 46.5%, *p* = 0.152) and 5-year DFS (48.6% vs. 36.3%, *p* = 0.098) of patients with poor immune-nutritional status (Figure 2). Moreover, univariate and multivariate Cox regression analyses showed that N stage and postoperative severe complications were the independent risk factors for OS within the subset of locally advanced ESCC patients with preserved immune-nutritional status. Furthermore, the risk factors for DFS included N stage, intraoperative hemorrhage, and SPCs (Table 4).

**Table 4.** Univariate and multivariate analysis of clinicopathological factors for OS and in high-PNI.

Characteristics	Overall survival				Disease-free survival			
	Univariable analysis		Multivariable analysis		Univariable analysis		Multivariable analysis	
	HR(95%CI)	*P*-value	HR(95%CI)	*P*-value	HR(95%CI)	*P*-value	HR(95%CI)	*P*-value
**Age(years)**								
≤65								
>65	1.426(0.943–2.156)	0.093			1.370(0.931–2.018)	0.111		
**Sex**								
Female								
Male	1.019(0.685–1.515)	0.927			0.892(0.612–1.300)	0.552		
**BMI (kg/m^2^)**								
≤18.5								
18.5–25	1.003(0.572–1.758)	0.992			1.258(0.707–2.235)	0.435		
≥25	1.217(0.609–2.432)	0.578			1.795(0.916–3.515)	0.088		
**Comorbidities**								
None								
Hypertension	0.688(0.394–1.201)	0.188			0.629(0.368–1.075)	0.09		
Diabetes	0.527(0.194–1.431)	0.209			0.570(0.233–1.392)	0.217		
Coronary heart disease	2.072(0.511–8.400)	0.308			1.526(0.377–6.176)	0.553		
**MUST**								
Low risk								
Medium risk	0.894(0.414–1.939)	0.777			0.822(0.400–1.688)	0.594		
High risk	1.451(0.935–2.252)	0.097			1.334(0.871–2.044)	0.185		
**ASA score**								
I/II								
III/IV	1.593(1.030–2.463)	0.036	1.267(0.793–2.025)	0.322	1.524(1.010–2.299)	0.045	1.174(0.754–1.828)	0.477
**Tumour location**								
Proximal								
Mid	1.320(0.735–2.367)	0.353			1.590(0.891–2.837)	0.117		
Distal	1.098(0.578–2.087)	0.776			1.320(0.704–2.474)	0.386		
**Histologic grade**								
Gx/G1								
G2	1.238(0.854–1.796)	0.26			1.189(0.842–1.681)	0.326		
G3	1.218(0.670–2.216)	0.518			1.164(0.675–2.008)	0.585		
**Tumour invasion**								
T1								
T2	0.997(0.417–2.389)	0.995			1.087(0.483–2.445)	0.84		
T3/T4a	1.698(0.790–3.649)	0.175			1.700(0.831–3.480)	0.146		
**Lymph node metastasi**s								
N0								
N1	1.575(0.985–2.518)	0.058	1.594(0.996–2.552)	0.052	1.563(1.018–2.398)	0.041	1.515(0.983–2.335)	0.060
N2	2.465(1.556–3.906)	<0.001	2.466(1.555–3.911)	<0.001	2.426(1.589–3.704)	<0.001	2.468(1.607–3.790)	<0.001
N3	5.534(2.967–10.322)	<0.001	5.978(3.186–11.218)	<0.001	5.369(2.923–9.862)	<0.001	5.450(2.928–10.145)	<0.001
**Neoadjuvant therapy**								
Yes								
No	0.826(0.522–1.307)	0.414			0.997(0.661–1.504)	0.988		
**Surgical method**								
McKeown								
Ivor Lewis	0.806(0.463–1.405)	0.447			0.849(0.512–1.408)	0.526		
**Lymphadenectomy**								
Two-field								
Three-field	0.859(0.508–1.454)	0.572			0.927(0.564–1.524)	0.765		
**Intraoperatve blood loss(ml)**								
≤100								
100–200	1.390(0.959–2.015)	0.082			1.229(0.863–1.750)	0.253	1.165(0.816–1.663)	0.401
≥200	1.630(0.950–2.795)	0.076			1.813(1.131–2.907)	0.013	1.915(1.177–3.114)	0.090
**Adjuvant therapy**								
No								
Yes	0.774(0.538–1.114)	0.167			0.688(0.489–0.969)	0.032	0.772(0.545–1.092)	0.144
**SPCs**								
No								
Yes	1.636(1.133–2.364)	0.009	1.653(1.111–2.460)	0.013	1.553(1.099–2.193)	0.013	1.462(1.009–2.119)	0.045

### 3.6. Establishment of nomograms based on SPCs in the preserved Immune-nutritional group

**Figure 3.**
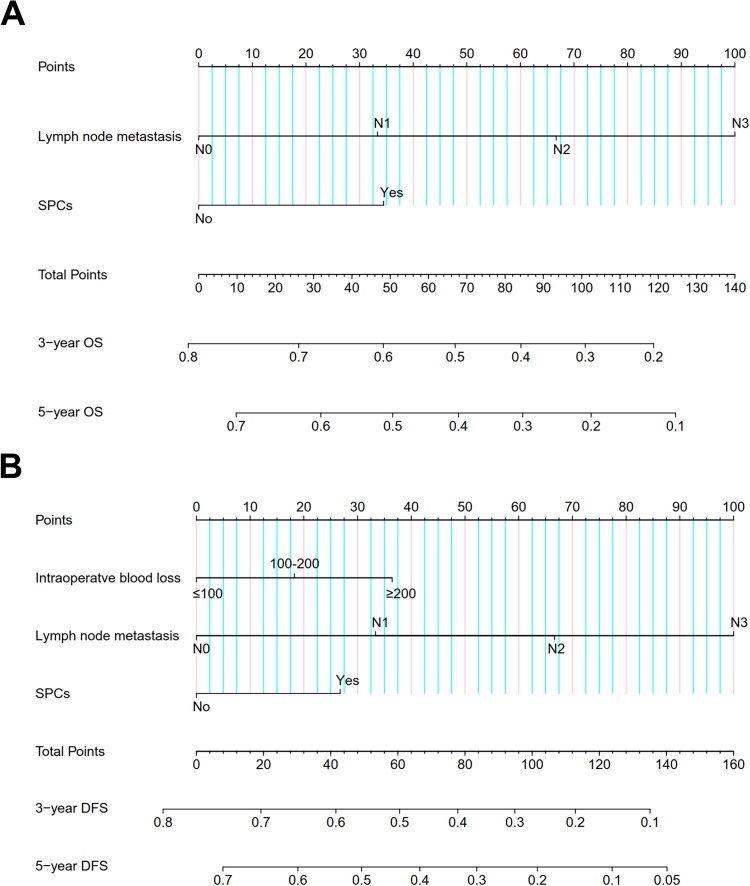
Nomogram for predicting OS (A) and DFS (B) in high-PNI.

**Figure 4.**
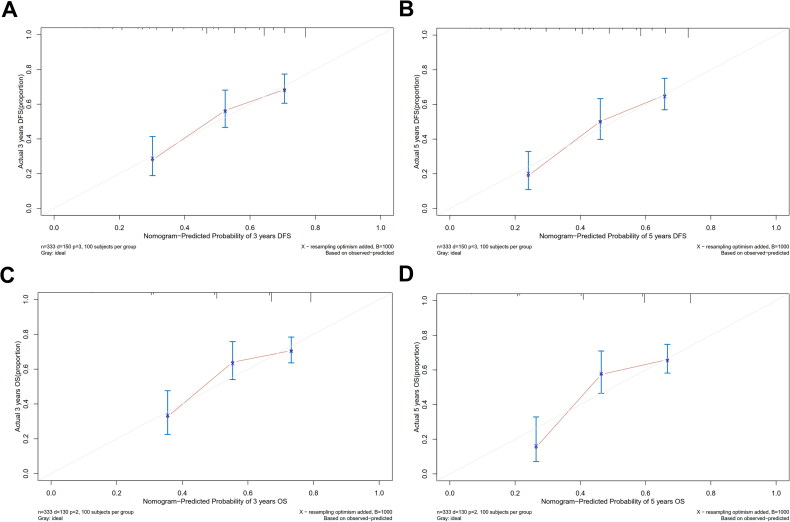
Calibration curves for 3-and 5-year OS and DFS based on nomogram in high-PNI.

A prognostic model was developed by incorporating the independent risk factors identified in the multifactorial Cox regression analysis into a nomogram to assess the risk of OS and DFS in locally advanced ESCC patients with preserved immune-nutritional status (Figure 3). The calibration curve exhibited a remarkable congruence between the prognostic predictions based on the nomogram and the actual clinical outcomes for 3-year and 5-year OS among patients with preserved immune-nutritional status. The model demonstrated a comparable consistency with clinical reality in predicting 3-year and 5-year DFS (Figure 4).

### 3.7. Risk staging for patients with preserved immunonutritional status

**Figure 5.**
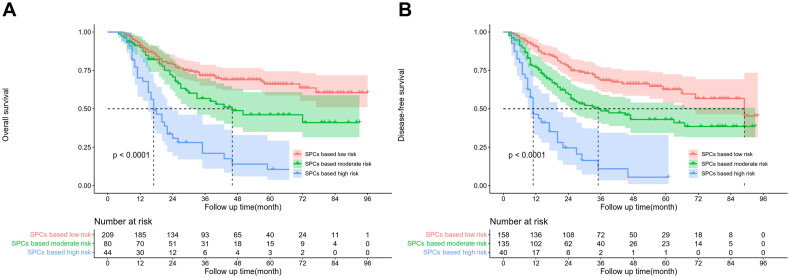
OS (A) and DFS (B) survival curves for low, intermediate, and high-risk patients stratified by nomogram score.

The X-Tile software was used for risk stratification of survival probabilities in locally advanced ESCC based on nomogram scoring. The patients were categorized into low-risk (<34 points), moderate-risk (34–67 points), and high-risk (>67 points) groups according to the optimal cut-off value for OS scoring (Figure S2). The 5-year OS rates for the low-risk, moderate-risk, and high-risk groups were 66.1%, 46.1%, and 10.5%, respectively (*p* < 0.001). Similarly, the patients were classified into low-risk (<45 points), moderate-risk (45–96 points), and high-risk (>96 points) groups based on the optimal cut-off value for DFS scoring (Figure S3). The 5-year DFS rates for the low-risk, moderate-risk, and high-risk groups were 62.5%, 43%, and 5.5%, respectively (*p* < 0.001) (Figure 5).

## 4. Discussion

This is the first study to elucidate the impact of SPCs on the long-term prognosis of patients with locally advanced ESCC after MIE based on preoperative immunonutritional status. Results showed that OS and DFS rates were significantly lower in the SPCs group than in the non-SPCs group for patients with preserved immunonutritional status. Furthermore, multifactorial regression analysis showed that SPCs are independent risk factors for OS and DFS. However, SPCs did not significantly affect the prognosis of patients with poor immunonutritional status.

Notably, there is controversy surrounding the impact of post-esophagectomy complications on the long-term survival of patients. Some studies have shown that patients who experience postoperative complications after esophageal cancer resection exhibit a poorer prognosis than those without such complications [7,23,24]. However, other reports have suggested that postoperative complications are not correlated with long-term survival rates after esophageal cancer resection [25,26]. In this study, SPCs significantly affected OS and DFS of patients with locally advanced ESCC after MIE. Nonetheless, the precise mechanisms by which SPCs affect the prognosis of esophageal cancer patients are unclear. Notably, several factors may be involved in this phenomenon. For example, Hirai et al. showed that surgical trauma induces excessive surgical stress, and the ensuing complications trigger a storm of inflammatory cytokine release during the perioperative period, increasing susceptibility to tumour metastasis, thus resulting in poor prognosis [27]. Residual cancer cells adhere to endothelial cells within blood vessels in the early postoperative phase when inflammatory cytokines are present, potentially elevating the chances of cancer metastasis and recurrence [28]. SPCs may lead to an adverse prognosis by increasing the likelihood of cancer metastasis and recurrence through the release of inflammatory cytokines, which exacerbate inflammation.

In this study, anastomotic leakage significantly affected locally advanced ESCC patients who have undergone MIE. Besides, previous studies showed that anastomotic leakage significantly affects the long-term survival of ESCC patients [29,30]. Anastomotic leakage is common after esophagectomy, and its occurrence is associated with an increased perioperative mortality rate. The postoperative onset of anastomotic leakage leads to the seepage of digestive fluids, causing local inflammation in milder cases, and may culminate in serious complications in severe cases, such as mediastinal abscess, empyema, and pneumothorax, thereby instigating a systemic inflammatory response. These findings indicate that SPCs may impact prognosis through a postoperative systemic inflammatory response. Moreover, studies have shown that inflammation is involved in cancer initiation and progression [31].

Meanwhile, postoperative pulmonary infections are prevalent after esophageal cancer radical surgery and are significantly related to patient prognosis. Studies have shown that various cytokines, such as IL-6, IL-8, and TNF-α, are highly expressed in bronchial epithelial cells. Notably, the occurrence of postoperative pulmonary infections is concomitant with the release of these inflammatory mediators. Tomohiko Nishi et al. showed that the upregulation of IL-8 and its receptor CXCR-2 is correlated with the progression and unfavourable prognosis of patients with ESCC post-esophagectomy [32]. Masayuki Yoneda et al. also indicated that IL-6 expression can mediate the invasion and metastasis of esophageal tumours, thereby promoting cancer development [33].

A diminished immune-nutritional status increases the risk of postoperative complications and is correlated with poorer postoperative survival [34]. In this study, OS and DFS were lower in the SPCs group than in the non-SPCs group for patients with favourable immunonutritional status. However, SPCs did not significantly affect the prognosis of patients with poor immunonutritional status. This may be due to PNI, which consists of serum albumin and lymphocyte counts, is associated with the prognosis of various types of cancer [16]. Some studies have reported that a range of inflammatory factors can cause lymphocytopenia, which promotes abnormal growth of tumour cells [35,36]. At the same time, serum albumin levels reflect the body’s nutrient reserves and reveal the severity of inflammation [37]. Therefore, low PNI reflects the state of immune deficiency and malnutrition of the patients. SPCs in patients will produce a large number of inflammatory factors, further reducing serum albumin and lymphocyte levels, thus inhibiting the anti-tumour effect. What’s more, Satoru Okada et al. showed that SPCs may impact prognosis after lung cancer surgery, particularly within the subset of patients with a well-preserved immunonutritional status [38]. Therefore, perioperative care is crucial for preventing SPCs in patients with poor immune-nutritional status and those with preserved immunonutritional status.

Clinical prognostic models have been widely used to evaluate the prognosis of various cancers, enabling personalized and precise predictions of patient-specific target outcome events. Unlike traditional staging, nomograms provide enhanced precision and are more easily understood by patients, thus aiding in clinical decision-making. In this study, the locally advanced ESCC patients with preserved immune-nutritional status were classified into low, intermediate, and high-risk groups based on the nomogram-derived scores for predicting OS and DFS. Notably, mortality and recurrence rates were significantly different among the three categories. These results indicate that nomogram scores can be used for risk stratification to provide a dependable prognostic system, representing a valuable supplement to the conventional TNM staging system. Clinicians should consider individualized close monitoring and effective adjuvant therapy for patients when the score indicates an increased risk of mortality or recurrence in ESCC patients.

Although this study had a large sample size, standardized surgical procedures, and standardized perioperative management, it has some limitations. First, this was a single-centre retrospective study that included a population of patients with ESCC from 2011 to 2018, so there was selection bias and chronology bias. At the same time, this study comes from a high-volume centre, which may limit the popularization and application of the nomogram. In addition, only ESCC patients were included in this study. Due to the differences in histology and clinical prognosis between ESCC and esophageal adenocarcinoma, the conclusions of this study may not be applicable to patients with esophageal adenocarcinoma. Moreover, there was heterogeneity in the surgical modalities included in this study, including Ivor-Lewis and McKeown esophagectomy, which may have contributed to relative performance bias. Currently, neoadjuvant therapy is recommended for patients with stage II/III ESCC. In our study, about 17.6% of the participants were on neoadjuvant therapy. Therefore, this may be a limitation in generalizing these results to other series. Finally, external validation is needed to assess the stability and applicability of the model since the predictive efficacy of nomograms can fluctuate with application contexts and demographics.

## 5. Conclusion

SPCs significantly reduce OS and DFS in ESCC patients based on PNI, especially for patients with preserved immunonutritional status. However, SPCs do not significantly affect the long-term survival of patients with poor immune-nutritional status. In this study, the developed nomogram exhibited good predictive ability. Moreover, the innovative staging system could effectively identify mortality and relapse risk in patients. Therefore, high-risk individuals among patients with advanced local ESCC with favourable immunonutritional status require individualized monitoring and proactive therapeutic interventions.

## Supplementary Material

Supplemental Material

## Data Availability

The data that support the findings of this study are available from the corresponding author, Shu-Chen Chen, upon reasonable request.
