# Supplementary material for: Severe postoperative complications after minimally invasive esophagectomy reduce the long-term prognosis of well-immunonutrition patients with locally advanced esophageal squamous cell carcinoma
Source: Ann Med. 2024 Dec 13;57(1):2440622. doi: 10.1080/07853890.2024.2440622 (PMC11648140; doi:10.1080/07853890.2024.2440622)

**Table S1.** Baseline characteristics of patients.

| Baseline variable | |  |  | (n=442) |
| --- | --- | --- | --- | --- |
|  |  |  |  |  |
| Age(years) | |  |  |  |
| ≤65 |  |  |  | 348(78.7%) |
| ＞65 |  |  |  | 94(21.3%) |
| Sex |  |  |  |  |
| Female |  |  |  | 133(30.1%) |
| Male |  |  |  | 309(69.9%) |
| BMI (kg/m2) | |  |  |  |
| ≤18.5 |  |  |  | 48(10.9%) |
| 18.5-25 |  |  |  | 338(76.5%) |
| ≥25 |  |  |  | 56(12.7%) |
| Comorbidities | |  |  |  |
| None |  |  |  | 358(81.0%) |
| Hypertension | |  |  | 65(14.7%) |
| Diabetes |  |  |  | 15(3.4%) |
| Coronary heart disease | | |  | 4(0.9%) |
| MUST |  |  |  |  |
| Low risk |  |  |  | 347(78.5%) |
| Medium risk | |  |  | 33(7.5%) |
| High risk |  |  |  | 62(14.0%) |
| ASA score | |  |  |  |
| I/II |  |  |  | 365(82.6%) |
| III/IV |  |  |  | 77(17.4%) |
| Tumor location | |  |  |  |
| Proximal |  |  |  | 44(10.0%) |
| Mid |  |  |  | 278(62.9%) |
| Distal |  |  |  | 120(27.1%) |
| Histologic grade | |  |  |  |
| Gx/G1 |  |  |  | 166(37.6%) |
| G2 |  |  |  | 217(49.1%) |
| G3 |  |  |  | 59(13.3%) |
| Tumor invasion | |  |  |  |
| T1 |  |  |  | 32(7.2%) |
| T2 |  |  |  | 79(17.9%) |
| T3/T4a |  |  |  | 331(74.9%) |
| Lymph node metastasis | | |  |  |
| N0 |  |  |  | 156(35.3%) |
| N1 |  |  |  | 148(33.5%) |
| N2 |  |  |  | 112(25.3%) |
| N3 |  |  |  | 26(5.9%) |
| Neoadjuvant therapy | | |  |  |
| Yes |  |  |  | 78(17.6%) |
| No |  |  |  | 364(82.4%) |
| Surgical method | |  |  |  |
| McKeown | |  |  | 396(89.6%) |
| Ivor Lewis | |  |  | 46(10.4%) |
| Lymphadenectomy | |  |  |  |
| Two-field |  |  |  | 391(88.5%) |
| Three-field | |  |  | 51(11.5%) |
| Intraoperatve blood loss(ml) | | |  |  |
| ≤100 |  |  |  | 223(50.5%) |
| 100-200 |  |  |  | 158(35.7%) |
| ≥200 |  |  |  | 61(13.8%) |
| Adjuvant therapy | |  |  |  |
| No |  |  |  | 259(58.6%) |
| Yes |  |  |  | 183(41.4%) |
| PNI |  |  |  |  |
| ≥47.1 |  |  |  | 325(73.5%) |
| ＜47.1 |  |  |  | 117(26.5%) |
| SPCs |  |  |  |  |
| No |  |  |  | 340(76.9%) |
| Yes |  |  |  | 102(23.1%) |

**Figure.S1** Postoperative Complications: Incidence and Types


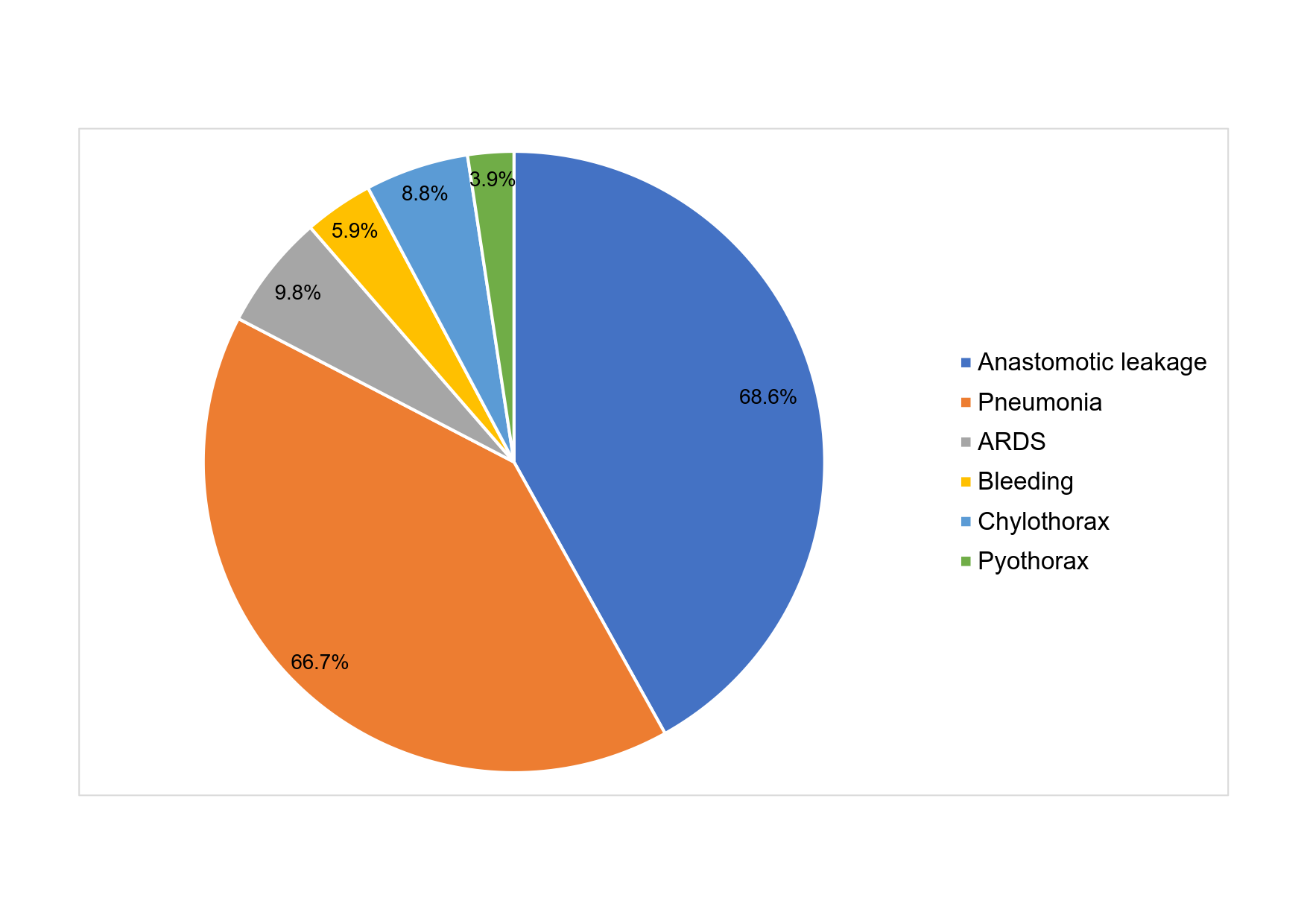


**Figure S2.** Best cutoff for nomogram scores for predicting OS


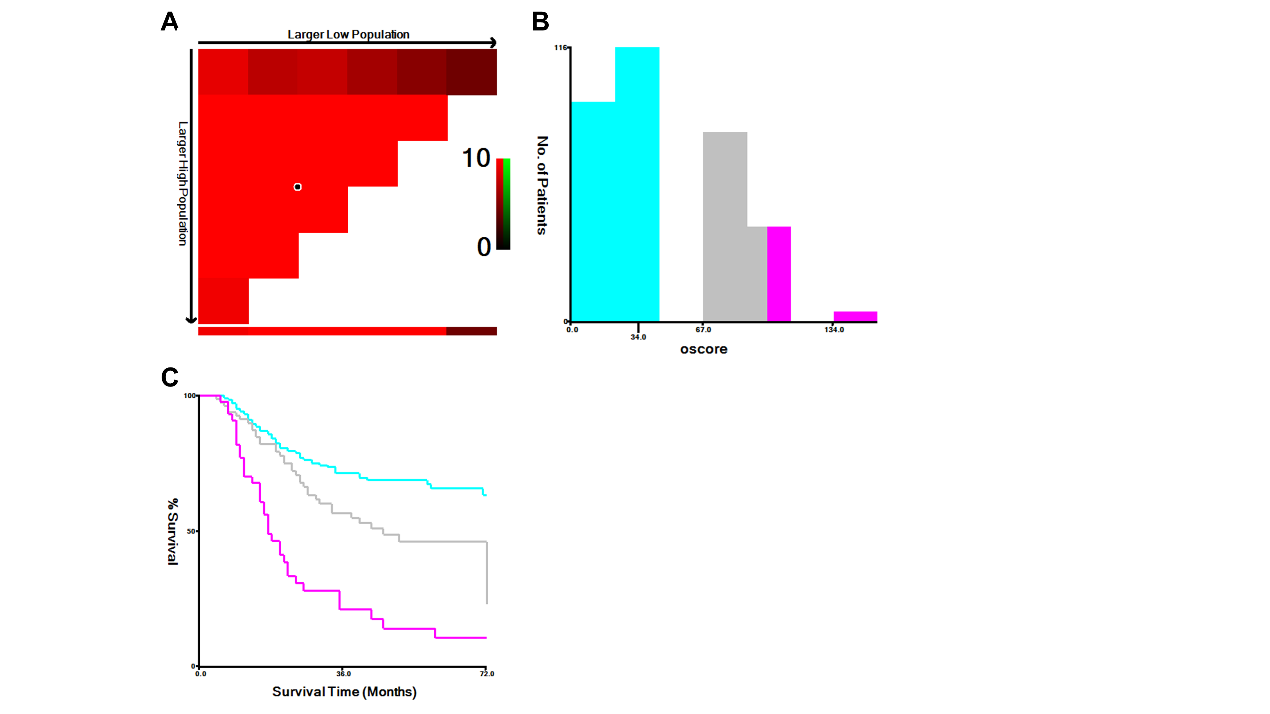


**Figure S3.** Best cutoff for nomogram scores for predicting DFS


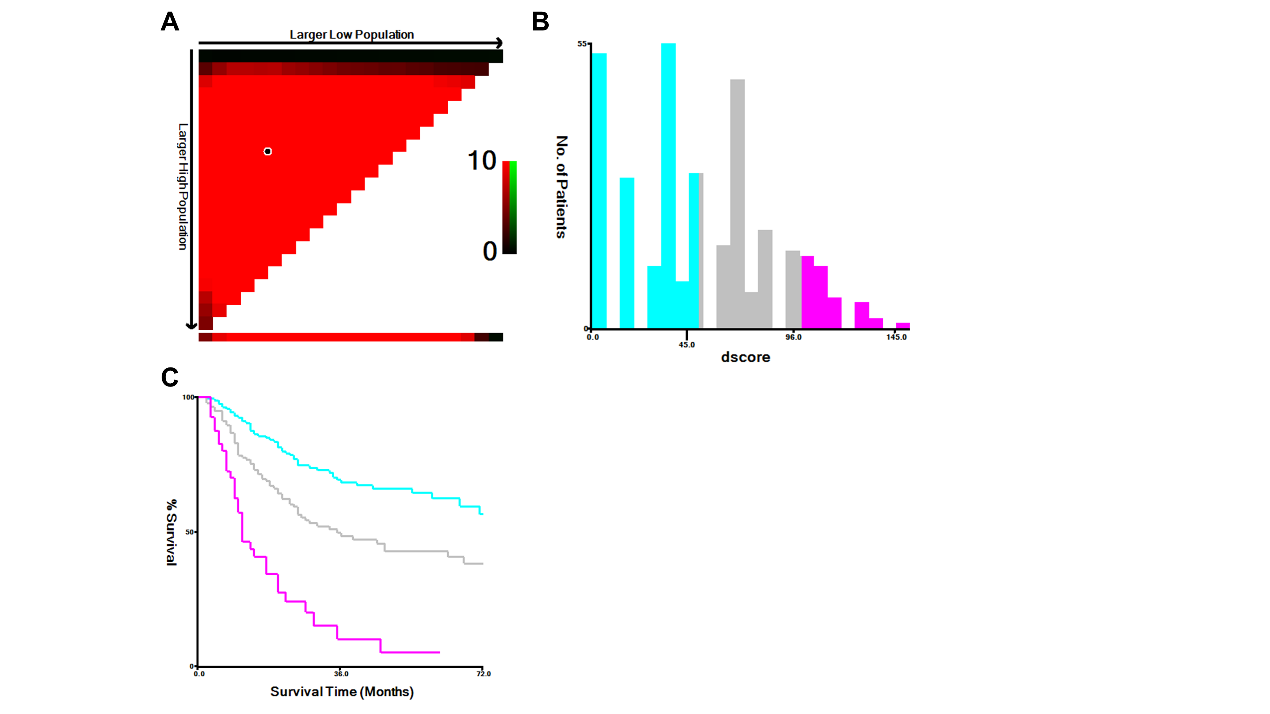

Supplement: Supplemental Material [file IANN_A_2440622_SM8548.docx]
